# Female Genital Mutilation/Cutting in the Swiss HIV Cohort Study: A Cross-Sectional Study

**DOI:** 10.1007/s10903-022-01390-0

**Published:** 2022-08-09

**Authors:** Fabio Mauri, Sara Cottler-Casanova, Matthias Cavassini, Marcel Stoeckle, Gilles Wandeler, Patrick Schmid, Dominique L Braun, Alexandra Scherrer, Enos Bernasconi, Alexandra Calmy, Jasmine Abdulcadir

**Affiliations:** 1 Department of Pediatrics, Obstetrics and Gynecology, Geneva University Hospitals, Bld de la Cluse, 1211, 0041-22- 3724049 Geneva, Switzerland; 2 Department of Epidemiology and Public Health, Swiss Tropical and Public Health Institute, Basel, Switzerland; 3 University of Basel, Basel, Switzerland; 4 Division of Infectious Diseases, Lausanne University Hospital, Lausanne, Switzerland; 5 Division of Infectious Diseases and Hospital Epidemiology, University Hospital Basel, Basel, Switzerland; 6 Department of Infectious Diseases, Bern University Hospital and University of Bern, Bern, Switzerland; 7 Division of Infectious Diseases and Hospital Epidemiology, Kantonsspital St. Gallen, St. Gallen, Switzerland; 8 Division of Infectious Diseases and Hospital Epidemiology, University Hospital of Zurich, Zurich, Switzerland; 9 Division of Infectious Diseases, Regional Hospital Lugano, Lugano, Switzerland; 10 HIV/AIDS Unit, Division of Infectious Diseases, Geneva University Hospitals, Geneva, Switzerland; 11 Institute of Medical Virology, University of Zurich, Zurich, Switzerland

**Keywords:** Female genital mutilation/cutting, FGM/C, HIV, Human immunodeficiency virus, Swiss HIV Cohort Study

## Abstract

FGM/C is a harmful practice that involves injury of the external female genitalia without medical purpose. It is mainly practiced in Africa, Asia, and the Middle East. However, with the migratory flows, women and girls with FGM/C and its consequences live all over the world. The lack of knowledge on how to care for women and girls living with FGM/C extends among all categories of health professionals involved in women’s health, including infectious disease specialists. This is a national, exploratory descriptive cross-sectional study aimed to generate descriptive statistics about FGM/C among HIV-infected migrant women included in the Swiss HIV Cohort Study (SHCS). Among the 387 women interviewed about FGM/C and who provided an answer, 80 (20.7%) reported to have undergone FGM/C. Fifty-six of the 80 women (70.0%) who reported having undergone FGM/C, also reported that they had never discussed their cutting with a health professional before. Our study demonstrates how common female genital mutilation is in women living with HIV and who have migrated to Switzerland and suggest how care and prevention could be improved significantly.

## Introduction

Female genital mutilation or cutting (FGM/C) is the practice of partial or total removal of the external female genitalia or injury to the genitals for non-medical reasons. The World Health Organization (WHO) classifies FGM/C into four types [1] (Table 1). This harmful practice has been documented in at least 27 African and 3 Asian countries and, as a result of migration, in high-income countries. The countries with the highest prevalence are Somalia (98%), Guinea (96%), Djibouti (93%), Egypt (91%), Eritrea (89%), Mali (89%), Sierra Leone (88%) and Sudan (88%) [2]. Estimates from the United Nations Children’s Fund (UNICEF) report that more than 200 million women and girls have undergone FGM/C globally [3] and that the number of migrants coming from FGM/C-practicing countries to high income countries will continue to increase [4]. Estimates from 2018 show that of the 36,898 women and girls living in Switzerland coming from one of the 30 high prevalence FGM/C countries, an estimated 21,706 have been cut or might be at risk of the practice [5]. However, indirect prevalence for women and girls living with FGM/C may not reflect the actual FGM/C prevalence among migrants in Switzerland and surveying samples of migrants might inform future estimates and care and prevention interventions.

**Table 1:** The WHO classification of female genital mutilation

Type I. Partial or total removal of the clitoral glans, and/or the prepuce/clitoral hood
Type Ia. Removal of the prepuce/clitoral hood only.
Type Ib. Removal of the clitoral glans with the prepuce/clitoral hood.
Type II. Partial or total removal of the clitoral glans and the labia minora, with or without removal of the labia majora
type IIa. Removal of the labia minora only.
type IIb. Partial or total removal of the clitoral glans and the labia minora
type IIc. Partial or total removal of the clitoral glans, the labia minora and the labia major
Type III. (Often referred to as infibulation). Narrowing of the vaginal opening with the creation of a covering seal. The seal is formed by cutting and repositioning the labia minora, or labia majora.
Type IIIa. Removal and repositioning of the labia minora.
Type IIIb. Removal and repositioning of the labia majora.
Type IV. All other harmful procedures to the female genitalia for non-medical purposes, for example pricking, piercing, incising, scraping and cauterization

Since the 1st of July, 2012, a specific article (art. 124) of the Swiss Penal Code states that it is an illegal and punishable offense «to mutilate the genitals of women or girls, impair their natural function considerably and lastingly or harm them in any manner» [6]. In 2016, the Swiss Network against Female Genital Cutting was created with the support of the Federal Office of Public Health [7]. As part of their activities, they conduct training of healthcare providers, healthcare students, certified interpreters and cultural navigators. Healthcare professionals are in fact not often trained about FGM/C and are therefore unaware of the different types of cutting, their possible negative health consequences and the existing management options. They also lack training on cultural, psychosexual, and legal information as well as counselling and prevention methods [8, 9]. Several studies have shown how such lack of knowledge is extended among all categories of health professionals involved in women’s health: gynecologists, pediatricians, general practitioners, infectious disease specialists, social workers, nurses and midwives [10–14]. This lack of training makes it difficult for health care providers to identify, diagnose and treat patients with FGM/C. A missed diagnosis of FGM/C can result in missed opportunities for the treatment and care of women as well as the prevention of FGM/C for her daughter(s) [15].

FGM/C can cause various negative health complications including adverse obstetric outcomes [16], long lasting psychological impact [17–19], painful vulvar scarring and pain, sexual dysfunction, genitourinary complications [17] [20] and infections, including the possibility of acquiring human immunodeficiency virus (HIV). The hypothesized mechanisms of HIV transmission related to FGM/C are the use of non-sterile equipment at the moment of the cutting, the need for blood transfusion in the event of hemorrhage and, increased risk of genital trauma due to vulvar scarring, with increased risk of sexually transmitted infections including HIV [21–24]. Some studies also show an association between FGM/C and HPV infection and cervical dysplasia [25], which might be worsened in case of an associated HIV infection. Some countries with a high prevalence of FGM/C, such as Ethiopia and Burkina Faso also register a high prevalence of HIV. [26, 27] Therefore, we hypothesize that infectious disease specialists, particularly HIV and travel medicine specialists regularly encounter women and girls potentially at risk or having already undergone FGM/C.

Our study aims to generate descriptive statistics about FGM/C among HIV-infected migrant women included in the Swiss HIV cohort study (SHCS). This will help to inform our estimates for the number of women living with FGM/C in Switzerland.

## Methods

This is a national, exploratory descriptive cross-sectional study. It has been approved by the Swiss Ethics Committees (Swissethics) and conducted according to the protocol, the Swiss legal requirements, the World Medical Association Declaration of Helsinki and the principles of Good Clinical Practice. The study was conducted in all Swiss Cantons after approval of the local ethical committees (2018 − 01851) and (01-142).

### Settings

The SHCS is a prospective cohort study with ongoing community and hospital enrolment of HIV-positive individuals in Switzerland. It has remained representative of the HIV patient population since its inception in 1988, and currently covers at least 75% of all patients receiving combination antiretroviral therapy (cART) and 69% of patients living with AIDS in Switzerland [28]. When an HIV infection is diagnosed, the concerned person is also informed of the existence of the SHCS and proposed to be enrolled and followed up by an infectious disease specialist at the main Swiss hospitals. If the person agrees, she signs a written and informed consent.

The number of migrant persons living with HIV (PLWH) is significant and has grown over time. However, their number is under-represented in the SHCS due to the trend of delaying access to health care HIV-related services in comparison with Swiss citizens or because it is easier to lose them to follow-up [29–31].

Prior to implementing the questionnaire, the Swiss infectious disease specialists were trained through a course on FGM/C that included information on the practice, cultural issues, countries at risk, complications, Swiss law and sites of referral to facilitate communication with the patients. Specific attention was paid to how to screen and discuss FGM/C in a culturally sensitive way. A PowerPoint presentation summarizing chapter two of the WHO clinical handbook for care of girls and women living with FGM (Communicating with girls & women living with FGM) was sent to all providers involved in administering the questionnaire [32]. The training was considered executed on the declaration of the specialists, without direct assessment by the authors.

### Participants

In this study, migrants are defined as non-Swiss citizens living on the Swiss territory for any length of stay regardless of permit or visa.

All women 18 years or above, originating from one of the 30 high-risk countries where FGM/C is practiced [29] were eligible. The questions were administered verbally by the patient’s physician during SHCS visits between June and December 2019 after written informed consent. In instances where language was a potential barrier, cultural mediators and certified interpreters were available without additional costs to the patients. For every affirmative response by a woman about whether she has undergone FGM/C, the healthcare provider would present the patient with information on counseling and healthcare services where she could be referred. The list of such services was provided together with the training PowerPoint presentation mentioned above.

### Measures

The routine SHCS questionnaire conducted every 6-months by the patient’s physician was enriched with the following additional questions over a 6-month period:

- Have you experienced female circumcision/genital cutting during your childhood?
- If the answer is yes; have you ever had the chance to discuss about it with a healthcare professional (doctor, nurse, etc.)?

The questions were developed by the principal investigators, submitted to the infectious disease specialists of the SHCS taking part to the training session, five adult patients of the outpatient clinic for women and girls with FGM/C and two certified interpreters of Geneva University hospitals to receive feedback before implementation.

### Analysis

The data was automatically extracted from the SHCS database and analyzed using an Excel spreadsheet.

## Results

The SHCS includes 9,690 people in active follow-up, of whom 5,471 hold Swiss citizenship (56.5%) and 4,219 (43.5%) hold a citizenship other than Swiss. Of 2675 women currently followed in the SHCS, 583 were considered for the two specific questions on FGM/C during the 6-month study duration based on their origin. Overall, 196 (33.6%) were not administered the questionnaire by the practicing physician for unknown reasons. Due to the fact that the data are anonymized except from the geographical origin of respondents, we could not discuss such result with the healthcare providers of the patients.

Among the 387 women interviewed about FGM/C and who provided an answer, 80 (20.7%) reported to have undergone FGM/C while 288 (74.4%) reported not having been cut. Nine women preferred not to answer and the remaining 10 did not understand the question according to the comment left by the interviewer. Fifty-six of the 80 women (70.0%) who reported having undergone FGM/C, also reported that they had never discussed their cutting with a health professional before.

Most women of the SHCS who reported living with FGM/C came from Eastern Africa, particularly Eritrea (41.3%) and Ethiopia (24.1%); followed by Ivory Coast (17.2%). The countries with the highest prevalence of FGM/C in the SHCS are Burkina Faso, Eritrea and Somalia which presents elevated rates of FGM / C in PLWH as expected, respectively of 31.3%, 41.3% and 62.5%. (Table 2).

**Table 2:** Participants’ origins

Nation	Total	History of FGM/C	missing
Central Africa			
Cameroon	193	2 (1.0%)	70
Central African Republic	3	1 (33,3%)	1
Guinea	9	2 (22.2%)	5
Eastern Africa			
Comore	1	0 (0.0%)	1
Eritrea	75	31(41.3%)	21
Ethiopia	58	14 (24.1%)	24
Kenya	67	2 (3.0%)	23
Somalia	8	5 (62.5%)	3
Sudan	1	1 (100.0%)	0
Tanzania	6	0 (0.0%)	2
Uganda	15	0 (0.0%)	5
Western Africa			
Benin	2	0 (0.0%)	0
Burkina Faso	16	5 (31.3%)	7
Ivory Coast	58	10 (17.2%)	20
Gambia	1	0 (0.0%)	0
Ghana	11	0 (0.0%)	6
Guinea-Bissau	5	2 (40.0%)	3
Liberia	2	0 (0.0%)	1
Niger	3	1 (33.3%)	1
Nigeria	16	3 (18.75%)	9
Senegal	6	1 (16.7%)	1
Togo	22	0 (0.0%)	12
Southeast Asia			
Indonesia	4	0 (0.0%)	0
Total	582	80 (13.7%)	215

The rate of missing questions is fairly evenly distributed with the origins of the patients, settling at around 30%, with peaks related to unrepresentative samples (for example 1 patient out of 1 in Comoros).

## Discussion and Conclusion

Our findings confirm that FGM/C is common in the SHCS patient population, even though our data may underestimate its presence given the large number of non-respondents.

20.9% of women in the SHCS said they had undergone FGM/C but in comparison to indirect prevalence estimates of FGM/C in Swiss university hospitals, the calculated prevalence according to ICD coding’s was 2.29% (95%CI: 1.98–2.62) [5]. This has been estimated as the proportion of the total number of FGM/C cases coded on the total number of women and girls from the targeted countries in four Swiss university hospitals between 2016 and 2018. This is a great example of how indirect estimates should be conducted alongside direct estimates as they may provide more accurate estimates that could guide policy- and clinical decision-making.

The strength of this study is the use of a standardized questionnaire (SHCS), employed since 1988 by healthcare professionals that are well trained in its administration, who received additional training for the questions on FGM/C for this study. A limitation is that we do not have information about the type and eventual complications of FGM/C among the participants. In our sample there are no Asian participants with FGM/C. Another limitation is related to the fact that the number of patients with FGM / C in our sample is probably underpowered for several factors. In addition to what is mentioned above, it is not possible to establish whether a woman with FGM/C reports that she has not been excised for fear or stigma, or because she does not remember because too young when it occurred.

Despite the research setting, a standardized questionnaire with only 2 binary questions to administer, and specific training for the healthcare professionals; more than one third of eligible participants were not interviewed about FGM/C. The reason for this lack of answers is not clear, but it can be deduced to be from the operator’s side as the patient’s refusal to answer was one of the possible alternatives. It is also possible that some awareness on epidemiology, complications and types of FGM/C are not enough when it comes to address, discuss and treat the issue of FGM/C and training should also focus on improving communication with women and girls with or at risk of having FGM/C.

It is difficult to understand why 10 people reported that they did not understand the question. It could be a problem of language barrier, of taboo linked to the topic (also considering the presence of interpreters in some cases) or of insufficient explanation by the physician. Additionally, more than two thirds of the included participants had never discussed their cutting with a health professional before. As shown in a previous study in France, where only 50% of general practitioners or travel doctors were aware of FGM/C and only 42.9% said they had seen it [11], our findings support the possibility that there is a lack of knowledge, practice and/or time among practicing physicians in charge of HIV patients (including gynecologist, infectious disease specialist, or general practitioners) when it comes to FGM/C. Although they might be used to asking questions about sexual and reproductive health, they may need more information and training on communication, screening, diagnosis, care, referral and prevention about FGM/C [10]. To plan appropriate future training interventions, it will be interesting to explore the barriers that prevent healthcare professionals from asking about genital cutting during consultations. Future research might also include men from countries with a high prevalence of FGM/C. They may have female partners and daughters who may be at risk or have experienced FGM/C and they could also benefit from discussing this subject with their health professional. Travel medicine specialists and general practitioners see families before travelling to home countries and discuss vaccines and malaria prophylaxis. These visits are a key moment to assess the eventual risk of FGM/C and take actions for prevention or protection from “vacation cutting”, which is when the child is taken abroad to undergo FGM/C [28]. However, both the lack of training, sensitization and limited time during visits makes FGM/C infrequently discussed and recorded [11].

There are lot of missed opportunities for discussing FGM/C with patients and, because of that, many opportunities of screening, detecting, treating and preventing FGM/C and its complications, particularly as genitourinary infections or cervical dysplasia in immunocompromised patients that might lead to worsened consequences [33].

FGM/C is common in the SHCS female population originating from Africa, but often overlooked by health professionals working with this population. Most women of the SHCS who reported living with FGM/C came from Eastern Africa, this is not surprising considering that more than 16,000 of the 36,898 migrant women from FGM/C high prevalence countries living in Switzerland in 2018 are originally from Eritrea [5]. Looking at the countries of origin, we can hypothesize that many of these women have undergone FGM/C types II and III which are frequent in Eastern and Western Africa, particularly in Eritrea, Ethiopia and the Ivory Coast. This is important as type II and particularly type III are the forms of cutting mostly associated with health complications including recurrent genitourinary infections [34, 35]. Recent studies have also suggested a possible association between FGM/C and cervical dysplasia and cancer, that again, in a female HIV positive population, deserves particular attention for screening and follow-up [25]. In FGM/C type III, defibulation (surgical opening of infibulation) could be proposed not only to treat eventual symptoms but also to facilitate cervical cancer screening and treatments [36].

FGM/C screening, diagnosis, care and prevention could be improved significantly through training and information. Various useful training material provided by the WHO [17] [32], other organizations [37] and some of the authors [38] are available. Training and continuing education courses are equally important for keeping up-to-date knowledge on this topic [39].

## Data Availability

The datasets used during the current study are available from the corresponding author on reasonable request.
